# Effect of fibrin glue as an adjuvant to hang-back surgery

**DOI:** 10.1186/1471-2415-12-14

**Published:** 2012-06-07

**Authors:** Jihyun Park, Jung Jin Lee, Eun Hye Lim, Joon H Lee, Kyung Hyun Jin, Ungsoo Samuel Kim

**Affiliations:** 1 Department of Ophthalmology, Kim’s Eye Hospital, Konyang University College of Medicine, Seoul, South Korea; 2 Myung-Gok Eye Research Institute, Konyang University College of Medicine, Seoul, South Korea; 3 Department of Ophthalmology, KyungHee University School of Medicine, Seoul, South Korea; 4 Department of Ophthalmology, Postgraduate School, Kyung Hee University, Hoegi-dong, Dongdaemun-gu, Seoul 130-701, Republic of Korea

**Keywords:** Fibrin glue, Hang-back surgery, Rabbit

## Abstract

**Background:**

The hang-back surgery is a useful technique in the field of strabismus surgery. The aim of this study is to determine the stabilizing effects of fibrin glue as an adjuvant to hang-back surgery.

**Materials and methods:**

Four (4)-mm hang-back recessions of the superior rectus muscle was performed in 32 eyes of 16 rabbits. Only in the left eye of the 16 rabbits, fibrin glue was applied between the recessed muscle bed and the sclera at the end of hang-back surgery (fibrin glue group). After 6 weeks, we compared the stability of the recessed rectus muscle between the fibrin glue group and the control group by evaluating the displacement of the muscle.

**Results:**

The frequency of stable insertion of the recessed muscle at the intended site was greater in the fibrin glue group (9 eyes) compared to the control group (3 eyes) (*p* = 0.028). In the control group, 5 eyes showed anterior displacement and 8 eyes showed posterior displacement and in the fibrin glue group, 1 eye showed anterior displacement, and 6 eyes showed posterior displacement. Anterior displacement was more common in the control group (6.3% Vs 31.3%). The control group and the fibrin glue group showed similar histological findings on microscopic examination.

**Conclusions:**

Fibrin glue is effective in stabilizing the new rectus muscle insertion and decreasing the displacement in the hang-back surgery.

## Background

Hang-back recession in strabismus surgery allows the extraocular muscle to hang on a suture loop from its original insertion site. This technique offers several advantages, including better surgical exposure and shorter operation time [1,2]. The risk of scleral perforation is also reduced, because the suture is passed through the thicker, more anterior sclera [3,4]. Animal studies, however, have shown that the hanging muscle could move horizontally or vertically before reattachment [5-9].

We implemented the use of fibrin glue in hang-back surgery. Fibrin glue is a tissue adhesive that forms a fibrin clot out of fibrinogen and thrombin. It has been used in various fields such as cardiovascular surgery and neurosurgery [10,11], and it has also been used effectively in ophthalmological procedures, including primary closure of corneal perforations, conjunctival closure, in managing of conjunctival wound leaks after trabeculectomy, and in the reattachment of extraocular muscles in rabbits or humans [12-19]. Fibrin glue as muscle sealant have been demonstrated to be strong enough in some reports but not in others [17-19]. We presumed that fibrin glue alone was not enough to attach the recessed muscle to the intended site, but it might help stabilizing the new insertion in hang-back recession. Therefore, the purpose of this study was to evaluate the capacity of fibrin glue to prevent displacement of the muscle reattachment after rectus muscle recession with hang-back surgery.

## Materials and methods

Thirty-two eyes of 16 pigmented rabbits (2.0 to 2.8 kg) were used in this study. Each rabbit was anesthetized with an intramuscular injection of xylazine hydrochloride (3 mg/kg; Rumpon®, Bayer, Monheim, Germany) and tiletamine/zolazepam (1.5 mg/kg; Zoletil®, Virbac, Carros, France) before the operation. An additional injection was given if necessary. All rabbits were handled, in accordance with the Institutional Animal Care and Use Committee of Konyang University.

**Figure 1:**
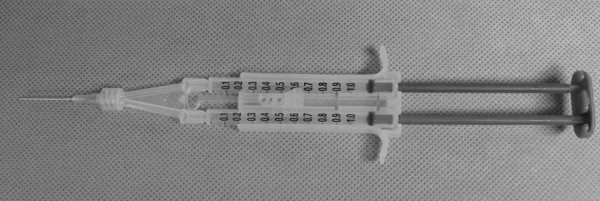
**Fibrin glue (Greenplast kit®).** Human fibrinogen concentrate and thrombin were loaded into each syringe and were simultaneously injected through Y-piece and application needle.

**Figure 2:**
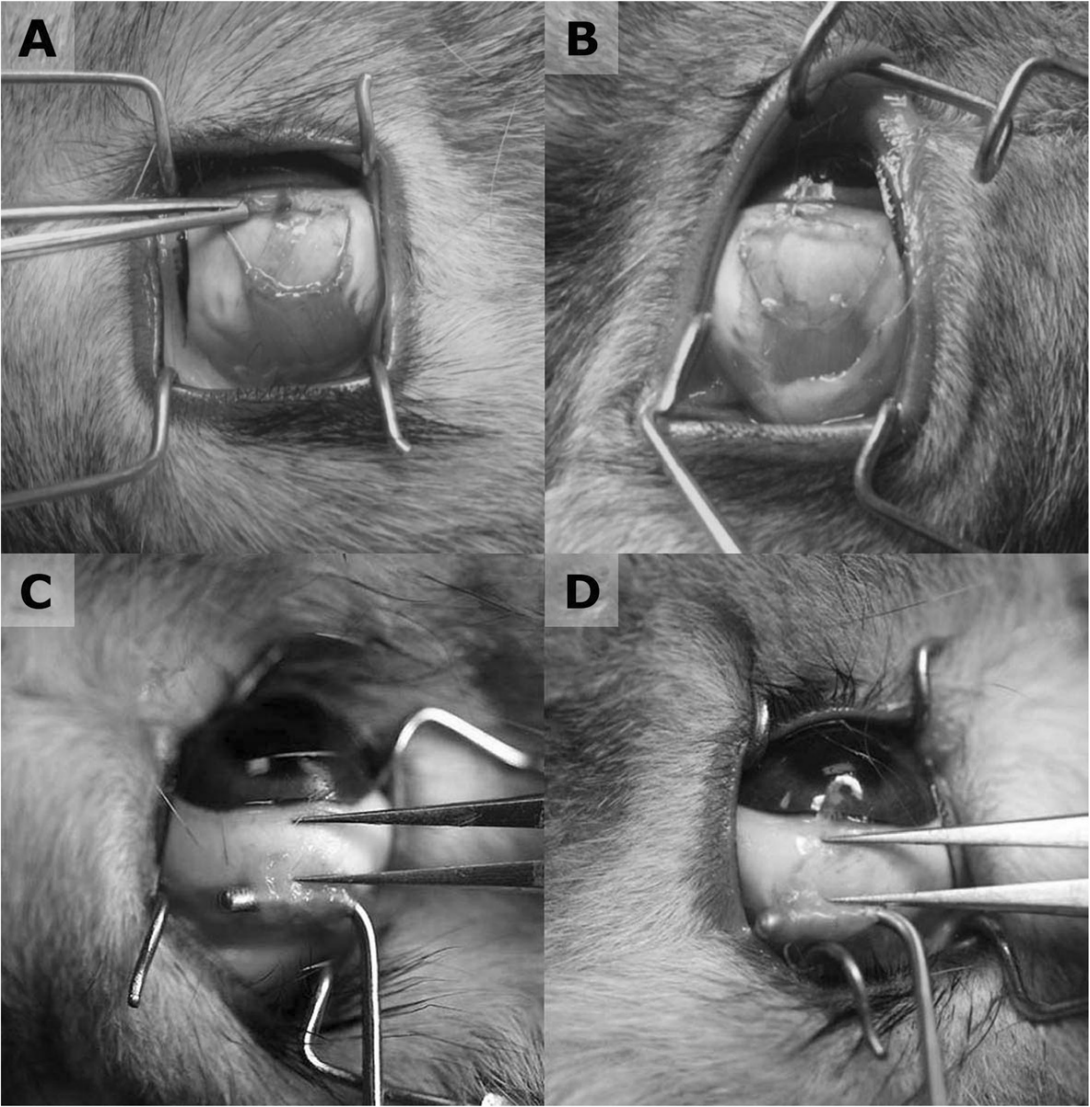
**Gross appearance immediately after (A,B) and 6 weeks after (C,D) surgery.** Conventional hang-back recession was performed on the superior rectus muscle of the both eyes, and one side was remained without any manipulation (A) and fibrin glue was applied between superior rectus muscle bed and the sclera only in the left eye (B) . After 6 weeks, the distance between the original and the new insertion site was grossly measured (C,D).

All surgeries were done by one skilled strabismus surgeon. The superior rectus muscle was exposed through a limbal incision, and two locking bites were placed at each edge of the muscle near its insertion site with double-armed 6–0 coated vicryl (Ethicon®, New Jersey, USA). After the muscle was separated from the globe, two needles were passed through each end of the original scleral insertion, with the needles pointing toward the center of the insertion site. The muscle was pulled back and placed 4 mm posterior to the original insertion site, and the knot was tied. Then in the right eye (control group), conjunctival suturing was done without an additional procedure, and in the left eye (fibrin glue group), small amount (about 0.02 ml: 0.01 ml from each syringe) of fibrin glue (Greenplast kit®, Green cross, Gyeonggi-do, Korea) was applied between the recessed muscle and the sclera. Human fibrinogen concentrate and thrombin were loaded into each syringe and were simultaneously injected through Y-piece and application needle (Figure 1). After a two-minute wait to allow for glue hardening, conjunctival suturing was done, as in the right eye (Figure 2a, b).

Six weeks later, the superior rectus muscles in all of the eyes were exposed through limbal incisions. In each eye, the distance from the center of the anterior border of the reattached muscle to the point just anterior to the original insertion site was grossly measured with calipers (Figures 2c, d). The anterior border of the new insertion was defined as the point just anterior to the engaged portion of the muscle by muscle hook. Anterior displacement was defined the final insertion was 0.5 mm or more anterior than the intended insertion and posterior displacement was the final insertion was 0.5 mm or more posterior than the intended insertion.

Four eyes from two randomly selected rabbits were enucleated and fixed in 10% neutral buffered formalin. Globes were dissected in the sagittal plane and routinely embedded in paraffin. Four-micrometer thick sections were stained with hematoxylin and eosin and studied under the light microscope.

We confirmed that the displacement of recessed muscle in 32 eyes of 16 rabbits followed a normal distribution using Kolmogorov-Smirnov test. The Fisher’s exact test and the Pearson chi-square test was applied to compare the stability of recessed muscle between the two groups—hang-back recession alone (control group) and hang-back recession in combination with fibrin glue (fibrin glue group) and *t*-test was used to compare the absolute value of the displacement. SPSS (version 15.0) was used for statistical analysis, with statistical significance defined as a p value less than 0.05.

## Results

The average distances from the original insertion site to the postoperative insertion site just after surgery were 3.94 ± 0.17 mm in the control group and 3.97 ± 0.22 mm in the fibrin glue group (*p* = 0.670) (Table 1). Six weeks after the surgery, some superior rectus muscles were displaced forward from the intended site, and some were displaced backward, in both groups. In the control group, five eyes (31.3%) showed anterior displacement (0.60 ± 0.22 mm, range 0.50 ~ 1.00 mm), and eight eyes (50.0%) showed posterior displacement (0.69 ± 0.26 mm, range 0.50 ~ 1.00 mm). In the fibrin glue group, one eye (6.3%) showed anterior displacement (0.5 mm), and six eyes (37.5%) showed posterior displacement (0.92 ± 0.38 mm, range 0.50 ~ 1.50 mm). The superior rectus muscle was attached to the intended site in three eyes in the control group (18.8%) and in nine eyes in the fibrin glue group (56.3%) (*p* = 0.028) (Table 2).

**Table 1:** Changes in distance from the original insertion site to postoperative insertion site 6 weeks after surgery

**Rabbit No.**	**Control group**			**Fibrin glue group**		
	**Distance at the end of surgery**	**Distance after 6 wk**	**Amount of displacement**	**Distance at the end of surgery**	**Distance after 6 wk**	**Amount of displacement**
1	4.0	3.5	−0.5	3.5	4.0	0.5
2	4.0	3.5	−0.5	4.0	4.0	0.0
3	4.0	3.0	−1.0	3.5	4.0	0.5
4	4.0	4.0	0.0	4.0	4.0	0.0
5	3.5	4.0	0.5	4.0	5.0	1.0
6	4.0	5.0	1.0	4.0	4.0	0.0
7	3.5	4.5	1.0	4.0	5.0	1.0
8	4.0	4.5	0.5	4.0	5.0	1.0
9	4.0	3.5	−0.5	4.0	4.0	0.0
10	4.0	5.0	1.0	4.0	4.0	0.0
11	4.0	3.5	−0.5	4.5	4.0	−0.5
12	4.0	4.5	0.5	4.0	4.0	0.0
13	4.0	4.5	0.5	4.0	4.0	0.0
14	4.0	4.0	0.0	4.0	4.0	0.0
15	4.0	4.0	0.0	4.0	5.5	1.5
16	4.0	4.5	0.5	4.0	4.0	0.0
						(mm)

**Table 2:** The number of cases and average distance of anterior displacement, stable insertion and posterior displacement of the recessed muscle

	**Control group**	**Fibrin glue group**	***p*-value**
Anterior displacement (mean, mm)	5 (0.60)	1 (0.50)	0.070^*^
No displacement	3	9	0.028^*^
Posterior displacement (mean, mm)	8 (0.69)	6 (0.92)	0.476^†^
Average amount of the absolute value of the displacement (mm)	0.53	0.38	0.333^‡^

* Fisher’s exact test.

† Pearson Chi-square test.

‡ t- test.

**Figure 3:**
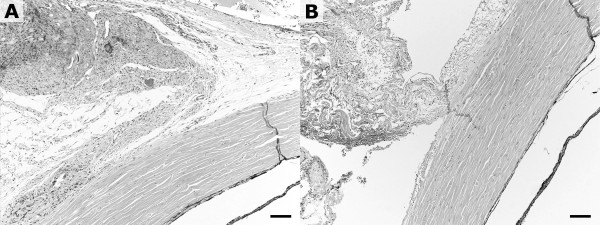
**Histological appearance 6 weeks after the surgery.** The right eye with conventional hang-back recession **(A)** and the left eye with fibrin glue after hang-back recession **(B**) showed similar findings. Rectus muscle at the insertion site was separated from the scleral surface and surrounded by fibrous tissue which was attached to the sclera. Inflammatory cells were rarely seen. Red blood cells were found more frequently in the right eye. Bar = 200 μm.

The control group and the fibrin glue group showed similar histological findings on microscopic examination. The sclera consisted of fine-textured collagen fibrous bundles, which were parallel to the surface of the globe. The collagen fibers in the left eye were partially cut off at the muscle insertion site, but they were nearly intact as a whole. The rectus muscle at the insertion site was separated from the scleral surface and surrounded by fibrous tissue, which was attached to the sclera. Residual fibrin glue or inflammatory cells were rarely seen (Figure 3b). The eye in the control group was not significantly different from the eye in the fibrin glue group, but it was characterized by more red blood cells (Figure 3a). The surrounding connective tissue didn’t show any significant inflammatory change.

## Discussion

There are chances of scleral perforation in the high risk situations such as thin sclera and restricted field of the operation, and in those cases, we can choose the modified suspension-recession technique or hang-back recession [20]. Several reports on hang-back recession in strabismus patients have shown that this technique is as effective as the conventional recession method [21,22]. However, [Capo et al. 23] reported somewhat different results, stating that the hang-back recession group had a significantly greater failure rate compared to the conventional recession group. They found that late over-corrections occurred more frequently in the hang-back recession group one year after surgery, and this is presumed to be a result of posterior displacement of the rectus muscle. [Wright 24] also described over-correction in small recessions, which resulted from central sag of hanging muscles.

Animal studies have shown the movement of hanging muscles both horizontally and vertically before being reattached in hang-back recession, with most of them exhibiting anterior displacement [5-8]. Several explanations have supported the pathogenesis of anterior displacement, including hemorrhage at the time of operation and direct forward growth of muscle fibers [5,6]. [Ohtsuki et al. 8] suggested that hang-back recession produces more connective fibrous tissue around the new insertion site compared to conventional recession and that this fibrous tissue might aid the muscle in growing forward.

In our study, the muscle moved, on the average, 0.16 mm backward in the control group and 0.31 mm backward in the fibrin glue group. At a glance, it can be seen that more displacement occurred in the fibrin glue group. However, it is reasonable to analyze both anterior and posterior displacement separately because both anterior and posterior displacements occurred in both groups. The results showed that anterior displacement occurred more frequently in the control group (5 of 16 eyes vs. 1 of 16 eyes), and so did posterior displacement (8 of 16 eyes vs. 6 of 16 eyes). Whatever the amount of recession is increased, especially in recession posterior to the equator, risk of anterior displacement would be increased. On the other hand, in low recession procedures, for example 4 mm recession, as in this study, risk of posterior displacement would be increased. And, only 3 eyes showed exact reattachment among 16 eyes with hang-back suture alone. The recessed muscle in the fibrin glue group, on the other hand, reattached with much more stability at the new insertion site (9 of 16 eyes), and the stability was statistically greater compared with the control group (*p* = 0.028). The recessed rectus muscle in the control group seemed to be movable in both forward and backward direction. Although explanations on such movements are not suggested in this study, the instability of hang-back technique alone might allow more room for unwanted under- or over-correction. Although posterior displacement may not be omitted because of sagging of posterior traction force of the recessed muscle, Fibrin glue creates an adhesive bed that prevent from anterior displacement. Thus, Fibrin glue may be helpful in stabilizing the new insertion in hang-back recession as expected.

It is, of course, quite another matter for fibrin glue to be used safely without sutures in strabismus surgery. [Tonelli et al. 18] reported on the instability of the sutureless Faden operation with fibrin glue, and [Spiere et al. 16] also showed that fibrin glue was not strong enough to overcome the contractive strength of the muscle in small amount of recessions. We did not measure horizontal displacement, however, the horizontal displacement would not be seen in small recession like 4 mm recession in the present study.

This combined surgical technique has several disadvantages. It takes a little more operation time for applying fibrin glue, costs more, and eliminates the possibility of performing adjustable suture surgery.

No significant complications were found in either group. Fibrin glue did not cause anticipated chemical problems; rather, it aided hemostasis, as shown in Figure 3. Whether this technique can be applied to human eyes, however, is a different matter. The composition of the extraocular muscles differs in rabbit eyes, as do eyeball movements and the severity of the postoperative inflammatory reaction [7,25]. Cost-effectiveness also has to be considered in clinical application.

## Conclusions

In summary, fibrin glue effectively stabilized the new rectus muscle insertion site after hang-back surgery by reducing the frequency of anterior and posterior displacement of the recessed muscle.

## Competing interests

The authors declare that they have no competing interest.

## Author’s Contributions

Authors: (JHP), (JJL), (EHL), (JHL), (KHJ), (USK) and USK carried out the animal studies, participated in the sequence alignment and drafted the manuscript. JHL carried out the histochemical stain and participated in the sequence alignment. JHP, KHJ and USK participated in the design of the study and performed the statistical analysis. JHP, KHJ and USK conceived of the study, and participated in its design and coordination and helped to draft the manuscript. All authors read and approved the final manuscript.

## Pre-publication history

The pre-publication history for this paper can be accessed here:

http://www.biomedcentral.com/1471-2415/12/14/prepub
